# Gene engineered mesenchymal stem cells: greater transgene expression and efficacy with minicircle vs. plasmid DNA vectors in a mouse model of acute lung injury

**DOI:** 10.1186/s13287-021-02245-5

**Published:** 2021-03-16

**Authors:** Maria Florian, Jia-Pey Wang, Yupu Deng, Luciana Souza-Moreira, Duncan J. Stewart, Shirley H. J. Mei

**Affiliations:** 1 Ottawa Hospital Research Institute, Ottawa, ON Canada; 2 The Ottawa Hospital, Ottawa, ON Canada; 3 University of Ottawa, Ottawa, ON Canada

**Keywords:** Mesenchymal stem cell, Acute lung injury, Minicircle, Acute respiratory distress syndrome

## Abstract

**Background:**

Acute lung injury (ALI) and in its severe form, acute respiratory distress syndrome (ARDS), results in increased pulmonary vascular inflammation and permeability and is a major cause of mortality in many critically ill patients. Although cell-based therapies have shown promise in experimental ALI, strategies are needed to enhance the potency of mesenchymal stem cells (MSCs) to develop more effective treatments. Genetic modification of MSCs has been demonstrated to significantly improve the therapeutic benefits of these cells; however, the optimal vector for gene transfer is not clear. Given the acute nature of ARDS, transient transfection is desirable to avoid off-target effects of long-term transgene expression, as well as the potential adverse consequences of genomic integration.

**Methods:**

Here, we explored whether a minicircle DNA (MC) vector containing human angiopoietin 1 (MC-ANGPT1) can provide a more effective platform for gene-enhanced MSC therapy of ALI/ARDS.

**Results:**

At 24 h after transfection, nuclear-targeted electroporation using an MC-ANGPT1 vector resulted in a 3.7-fold greater increase in human ANGPT1 protein in MSC conditioned media compared to the use of a plasmid ANGPT1 (pANGPT1) vector (2048 ± 567 pg/mL vs. 552.1 ± 33.5 pg/mL). In the lipopolysaccharide (LPS)-induced ALI model, administration of pANGPT1 transfected MSCs significantly reduced bronchoalveolar lavage (BAL) neutrophil counts by 57%, while MC-ANGPT1 transfected MSCs reduced it by 71% (*p* < 0.001) by Holm-Sidak’s multiple comparison test. Moreover, compared to pANGPT1, the MC-ANGPT1 transfected MSCs significantly reduced pulmonary inflammation, as observed in decreased levels of proinflammatory cytokines, such as tumor necrosis factor-alpha (TNF-α), interferon-gamma (IFN-γ), interleukin-6 (IL-6), monocyte chemoattractant protein-1 (MCP-1), and macrophage inflammatory protein-2 (MIP-2). pANGPT1-transfected MSCs significantly reduced BAL albumin levels by 71%, while MC-ANGPT1-transfected MSCs reduced it by 85%.

**Conclusions:**

Overall, using a minicircle vector, we demonstrated an efficient and sustained expression of the ANGPT1 transgene in MSCs and enhanced the therapeutic effect on the ALI model compared to plasmid. These results support the potential benefits of MC-ANGPT1 gene enhancement of MSC therapy to treat ARDS.

## Introduction

Acute respiratory distress syndrome (ARDS) was first described five decades ago in patients with tachypnea and refractory hypoxemia, with main symptoms consisted of diffuse opacities on chest radiographs [1]. Common causes of ARDS include pneumonia, trauma, multiple transfusions, severe burns, and septic shock. The disease incidence ranges from 10 to 86 cases per 100,000 per year [2], with hospital mortality in those with severe ARDS at 46% [3]. Many survivors have persistent pulmonary dysfunction, skeletal-muscle weakness, and reduced health-related quality of life [4, 5]. Lung-protective ventilation is employed to provide support for the failing respiratory system [6]; however, patients diagnosed with ARDS still have limited treatment options and poor prognosis.

Mesenchymal stem cell (MSC) therapy is considered a promising intervention for treating ARDS due to the immunomodulatory and anti-inflammatory ability of these cells [7, 8]. Previous studies have demonstrated that bone marrow-derived mesenchymal stem cells (BM-MSCs) can reduce pulmonary inflammation and lung permeability [9–11]. The immunomodulatory capacity of MSCs is believed to be largely mediated through the secretion of paracrine/endocrine factors and/or direct interaction with host immune cells [12, 13]. There are ongoing translational efforts to study the safety and efficacy of MSCs in patients with ARDS, including the recently completed START [14, 15] and MUST-ARDS trials [16]. More recently, in rapid response to the COVID-19 pandemic, MSCs are being deployed in human trials studying SARS-COV2-induced ARDS [17]. For example, Leng et al. have reported improved pulmonary function and symptoms of COVID-19 patients who received MSC therapy [18].

We have previously reported improved therapeutic efficacy of MSCs that can be enhanced by plasmid DNA angiopoietin-1 transfection (pANGPT1) in LPS-induced ALI in mice [9]. As a vascular protective factor, ANGPT1 reduces endothelial permeability and inhibits leukocyte-endothelium interactions by modifying endothelial cell adhesion molecules and cell junctions [19–22]. However, plasmid bacterial sequences induce innate immune responses that limit the amount and duration of transgene expression and can exacerbate inflammation due to inherent bacterial CpG content [23]. Minicircle DNA vectors consist of a circular expression cassette devoid of the bacterial plasmid DNA backbone, which results in less immunogenicity and more sustained transgene expression in quiescent cells/tissues [24, 25]. Serra et al. demonstrated that MSCs transfected with minicircles have higher VEGF expression than plasmid-based transfected cells using an in vitro angiogenesis model [26]. Additionally, Mun et al. showed that minicircle delivery via microporation significantly improved transfection efficiency compared to the cationic liposome system [27]. Herein, we compared the minicircle DNA system to a conventional plasmid vector for the transfection of MSCs and examined the potential impact on their therapeutic benefit in an ALI murine model.

## Materials and methods

### Characterization, culture, and transfection of MSCs

**Fig. 1:**
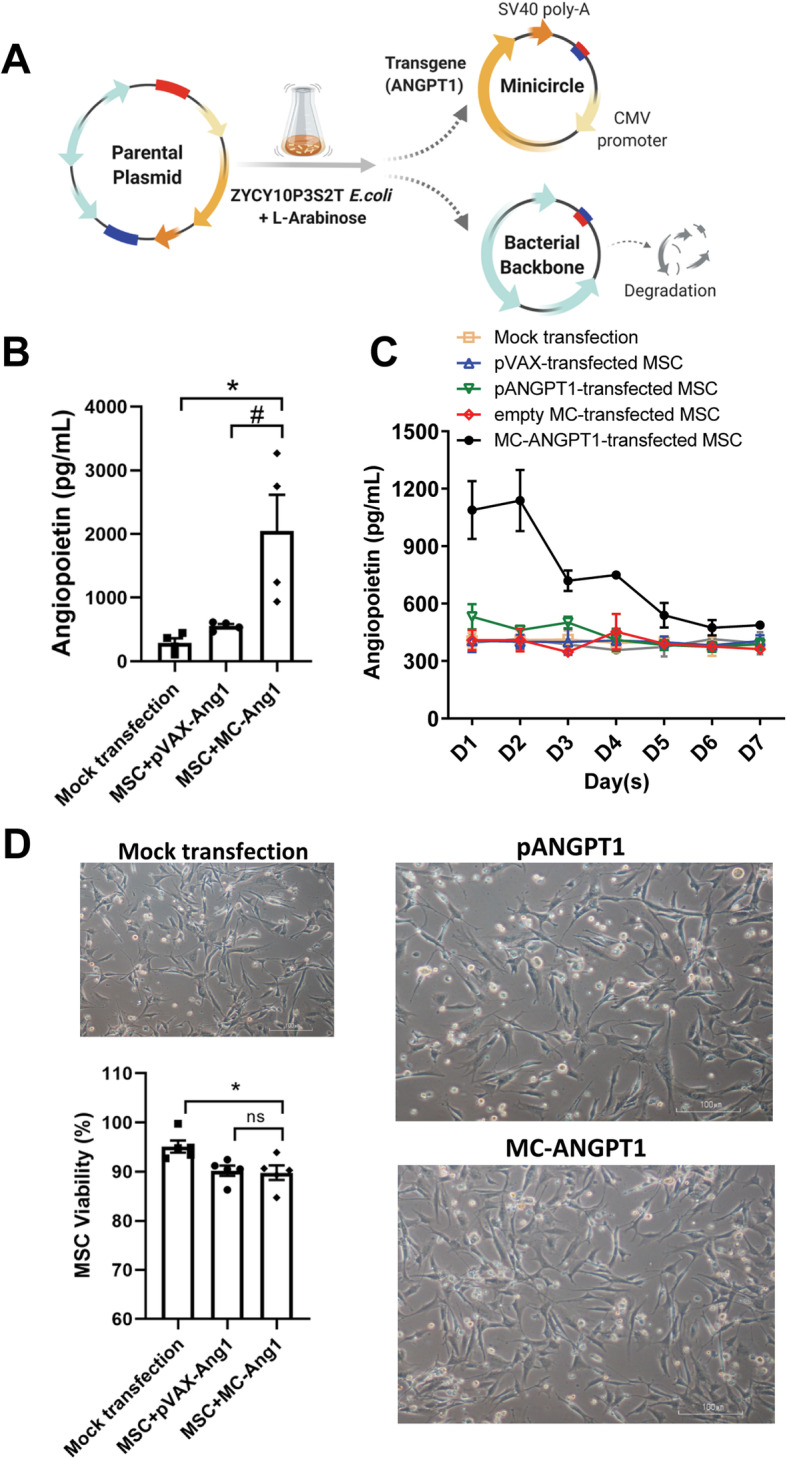
Comparison of plasmid or MC-based transfection in mouse MSCs. **a** Schematic illustration of method in generating minicircles (adapted from www.systembio.com). **b** Nuclear-targeted electroporation (Amaxa) of MC-ANGPT1 resulted in a 3.7-fold increase in human ANGPT1 protein in conditioned media using ELISA (R&D Systems) at 24 h after transfection, compared to the same amount of plasmid ANGPT1 (pANGPT1). *n* = 4 experiments. **c** MSCs transfected with nucleofection using reagent only (mock), empty plasmid (pVAX), human ANGPT1 plasmid (pANGPT1), empty minicircle DNA (MC), or human ANGPT1 minicircle DNA (MC-ANGPT1). MC-ANGPT1 peak values at D1 and D2 compared to pANGPT1 and mock transfected. *n* = 2 experiments. **d** MSC viability evaluated by trypan blue exclusion (bar graph) and cell morphology (photomicrographs) at 24 h after transfection. *n* = 5 experiments. Group comparisons were analyzed by one-way ANOVA with Holm-Sidak’s post hoc test. *p < 0.05, mock-transfection vs. MC-ANGPT1-transfected MSCs. #*p* < 0.05, pANGPT1-transfection versus MC-ANGPT1-transfected MSCs

A frozen vial of murine MSCs (isolated from male C57Bl/6J mice; courtesy of Dr. Darwin Prockop, Texas A&M HSC COM, Temple, TX, USA) was thawed and expanded as previously described [28]. Differentiation and characterization of MSCs was evaluated using a Mesenchymal Stem Cell Functional Identification Kit (R&D Systems) as per the manufacturer’s instructions and described in previous publication [9, 28]. The full-length coding sequence of human *ANGPT1* (1115 bp) was cloned into the expression vector pVAX-CMV-1 (Sigma) as previously described [29]. For minicircle (MC)-ANGPT1-1 cloning, the full-length coding sequence templet ANGPT1/pENTR223.1 was used. The ZYCY10P3S2T *Escherichia coli* in the presence of Kanamycin 50 μg/ml was used for minicircle production. Competent bacteria were inserted with parental CMV-ANGPT1 to generate MC-ANGPT1 by addition of 20% L-arabinose. Using the Minicircle Production Strain approach, the bacterial backbone was excised and degraded (SBI technology) (Fig. 1a). The circular DNA fragments from the parental plasmid (Minicircle) were extracted using the EndoFree Plasmid Maxi Kit (Qiagen). MSCs were transfected by nuclear-targeting electroporation using nucleofection (Amaxa, Lonza). MSCs were nucleofected with empty plasmid (pVAX), human ANGPT1 plasmid (pANGPT1), empty minicircle DNA (MC), and human ANGPT1 minicircle DNA (MC-ANGPT1). Cells receiving only the transfection reagent were used as transfection control (mock). To evaluate ANGPT1 protein expression over time, media was changed daily and stored for further analysis by ELISA (R&D Systems), following manufacturers’ recommended protocols. Cell viability was evaluated by trypan blue exclusion.

### Murine model of LPS-induced ALI

All animal procedures were approved in advance by the Animal Care Committee of the University of Ottawa (Ottawa, Ontario, Canada, protocol number OGH/RI-42). C57Bl/6J female mice (10–12 weeks) were anesthetized with 120 mg/kg of ketamine with 6 mg/kg of xylazine. Acute lung injury was induced by intratracheal instillation of 5 mg/kg of LPS (100 μg/mouse, *E. coli* 055:B5; Sigma) dissolved in 30 μL of normal sterile saline. For animal treatment, we used MSCs harvested 24 h after transfection. A suspension of 2.5 × 10^5^ cells MSC in PBS (100 μl total volume) was slowly infused into each mouse via a jugular venous cannula 30 min following LPS challenge.

After infusion, the cannula was withdrawn, the vein ligated and the incision sutured using silk suture. Mice were then sacrificed 3 days after LPS to evaluate the therapeutic efficacy by collecting tissues for analysis. Bronchoalveolar lavage (BAL) fluid was obtained by inserting a catheter into the trachea and slowly injecting and aspirating 1 mL of saline three times. Cells and fluids obtained from BAL were used for analysis. Total cell counts were determined using a hemocytometer. BAL was then centrifuged at 800*g* for 10 min at 4 °C and the supernatant was collected and stored at − 80 °C for further analysis; cell pellet was resuspended and cytospun onto slides. Differential cell counts were determined on BAL cytospin slides stained with Hemacolor (EMD Chemicals). The number of neutrophils was calculated as the percentage of neutrophils multiplied by the total number of cells in the BAL fluid sample. All analyses were performed in a blinded fashion.

### Measurement of albumin, IgM, cytokines, and chemokines

Albumin and IgM levels in BAL fluid samples were measured using a murine-specific albumin ELISA kit (ALPCO Diagnostics) and a murine-specific IgM ELISA kit (Bethyl Laboratories), following the manufacturers’ recommended protocols, respectively. Cytokine and chemokine levels (IFN-γ, TNF-α, IL-6, IL-1β, MIP-2, and MCP-1) in BAL fluid were measured by multiplex Luminex immunoassays kit (Bio-Rad).

### Statistics and software

Data in figures are represented as individual data points in a scatter plot with a bar to indicate the mean. Differences between the treated groups versus the injured group (LPS/PBS) were assessed using a one-way ANOVA (with post hoc comparisons using Bonferroni or Holm-Sidak’s test) with statistical software (GraphPad Prism version 8). A value of *p* < 0.05 was considered statistically significant. Illustrations of generating minicircles were created using BioRender.com.

## Results

### Minicircle DNA-transfected MSCs released significantly more ANGPT1 proteins compared to plasmid transfection

Significantly higher levels of ANGPT1 proteins were detected in conditioned media of MSCs transfected with ANGPT1 coding sequence using minicircle (MC-AGNPT1) vs. plasmid (pANGPT1) vectors at 24 h post transfection (2048 ± 567 pg/mL vs. 552.1 ± 33.5 pg/mL, respectively; Fig. 1b). ANGPT1 daily production was highest at day 1 and 2 after MC-ANGPT1 transfection, followed by a reduction at day 3 to 719.6 ± 54.3 pg/mL and continued to decrease by day 7 to 487.2 ± 22.5 pg/mL, with an empty MC vector at 361.4 ± 18.4 pg/mL, or with mock transfection at 384.6 ± 19.6 pg/mL (Fig. 1c). MSCs transfected with MC-ANGPT1 and pANGPT1 showed similar morphology and viability at 24 h after transfection (90.2 ± 2.3 vs 89.8 ± 3.3; Fig. 1d). Overall, our data showed transfection in MSCs using minicircles can result in a higher level of ANGPT1 protein overexpression compared to using plasmid.

### Overexpression of ANGPT1 with MC-DNA vector transfection strategy enhanced therapeutic efficacy of MSCs in an animal model of acute lung injury

**Fig. 2:**
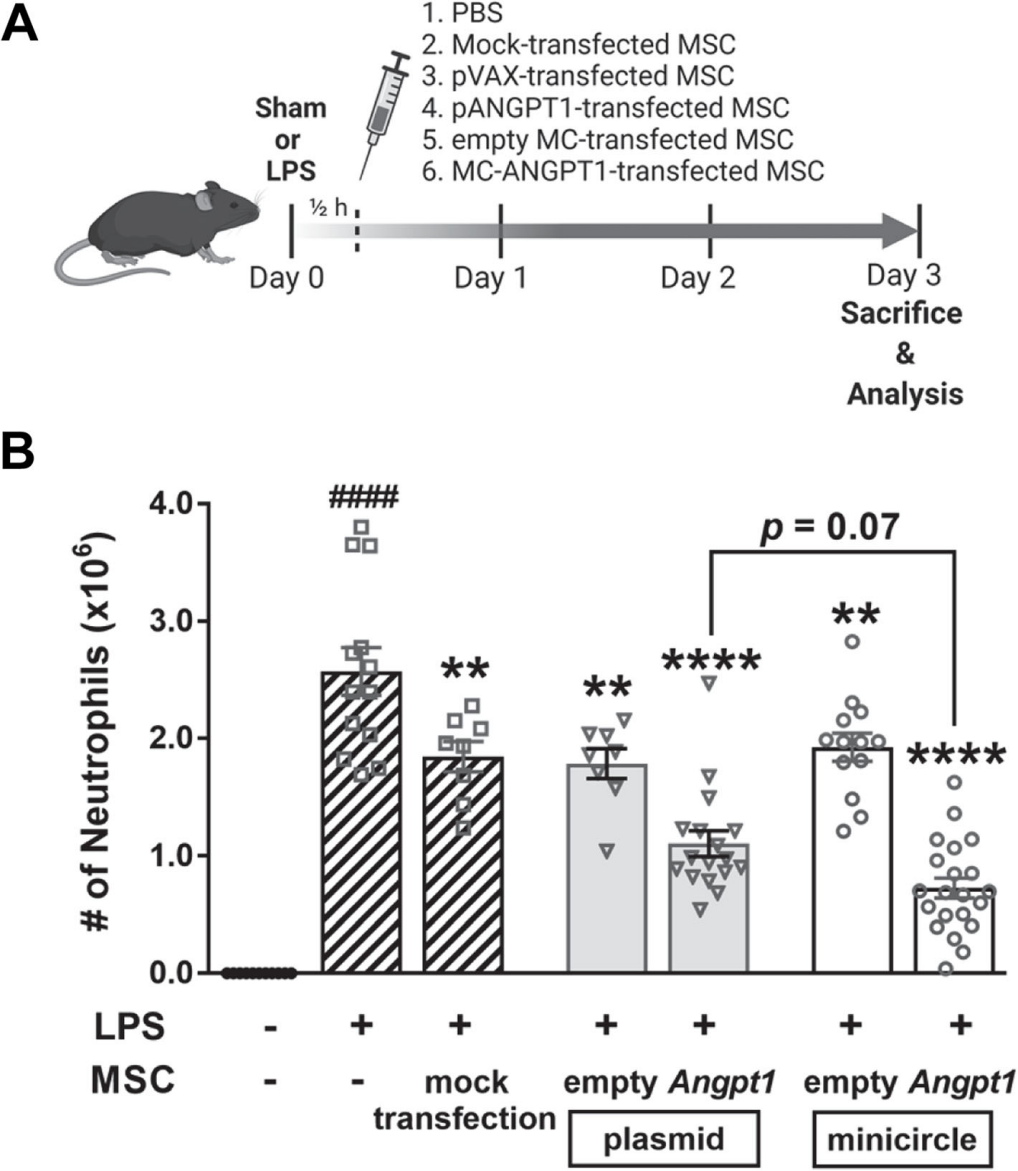
Therapeutic efficacy of MSCs alone or transfected with ANGPT1 in reducing LPS-induced lung inflammation in mice. **a** Experimental design for in vivo study. C57Bl/6J mice were first challenged by intratracheal instillation of LPS, followed by respective treatment after 30 min. Mice were then sacrificed 3 days after LPS to evaluate therapeutic efficacy. **b** Number of neutrophils in bronchoalveolar lavage (BAL) fluid were obtained from differential counts via cytospin to evaluate lung airspace inflammation. Group comparisons were analyzed by one-way ANOVA with Bonferroni post hoc test. ^####^*p* < 0.0001, sham/vehicle versus LPS/vehicle group. **p < 0.01 and ****p < 0.0001, LPS/vehicle vs. each treated group

Acute lung injury was induced by intratracheal LPS challenge in mice (Fig. 2a), followed by treatment with PBS or MSCs (5 groups) at 30 min, and mice were then sacrificed at 3 days after LPS. Lung airspace inflammation, evaluated by the number of neutrophils in bronchoalveolar lavage (BAL), was significantly reduced in all MSC-treated mice (MSCs transfected with empty plasmid or MC, pANGPT1, or MC-ANGPT1 compared to LPS/PBS group), while mice receiving MC-ANGPT1-transfected MSCs showed a strong trend towards reduced lung inflammation compared to pANGPT1-transfected MSCs (*p* = 0.07) (Fig. 2b).

### Overexpression of ANGPT1 using MC-DNA vector significantly reduced pulmonary inflammation compared to plasmid-based strategy

**Fig. 3:**
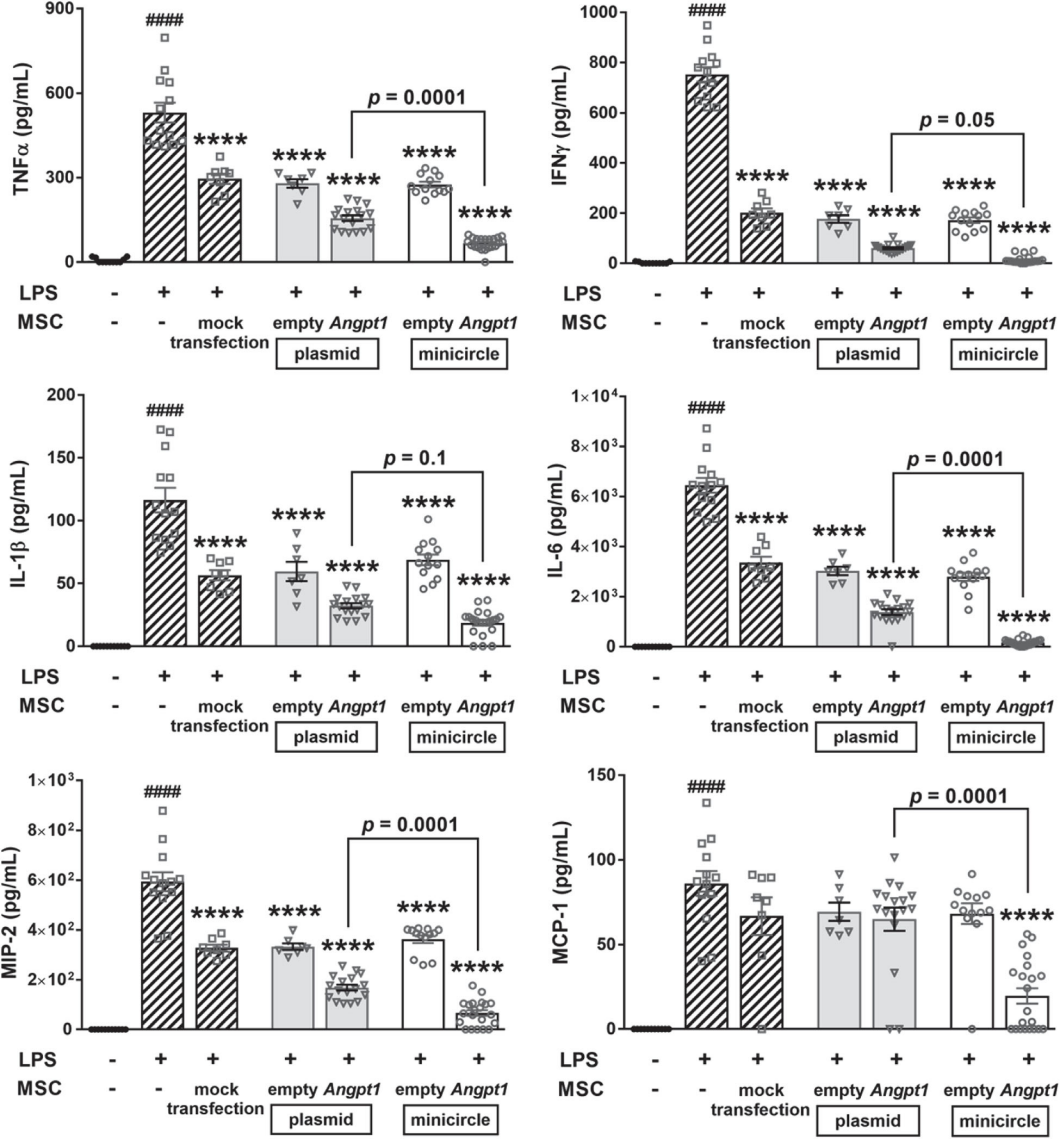
Effect of MSCs alone or transfected with ANGPT1 (plasmid vs. MC) on LPS-induced pulmonary inflammation. Pulmonary inflammation was assessed by measurement of multiple cytokines in BAL fluids using multiplex luminex immunoassays kit (Bio-Rad). Group comparisons were analyzed by one-way ANOVA with Bonferroni post hoc test. ^####^*p* < 0.0001, sham/vehicle vs. LPS/vehicle group. ****p < 0.0001, LPS/vehicle vs. each treated group

We next assessed pulmonary inflammation caused by LPS-induced ALI by measuring the levels of inflammatory cytokines in BAL fluids. Proinflammatory cytokines, such as TNF-α, IFN-γ, IL-1β, IL-6, MCP-1, and chemokine MIP-2, were all significantly elevated in BAL fluid of mice in the LPS/vehicle group in response to LPS exposure, compared to naïve animals receiving a vehicle only (Fig. 3). Treatment with MSCs (with mock transfection, empty plasmid or empty minicircles) decreased the level of proinflammatory cytokines, while transfection with MC-ANGPT1 decreased cytokine levels significantly to levels close to the baseline values observed in naïve mice (*p* < 0.0001, LPS/vehicle versus pANGPT1- or MC-ANGPT1-transfected MSC-treated groups). Furthermore, MC-ANGPT1-transfected MSCs resulted in significantly greater reduction in pulmonary cytokine levels compared to pANGPT1-transfected MSCs (*p* = 0.0001 for TNF-α, *p* = 0.05 IFN-γ, *p* = 0.0001 IL-6, *p* = 0.0001 MCP-1, and *p* = 0.0001 MIP-2; Fig. 3).

### Overexpression of ANGPT1 using MC-DNA vector was significantly more effective in reducing pulmonary vascular permeability

**Fig. 4:**
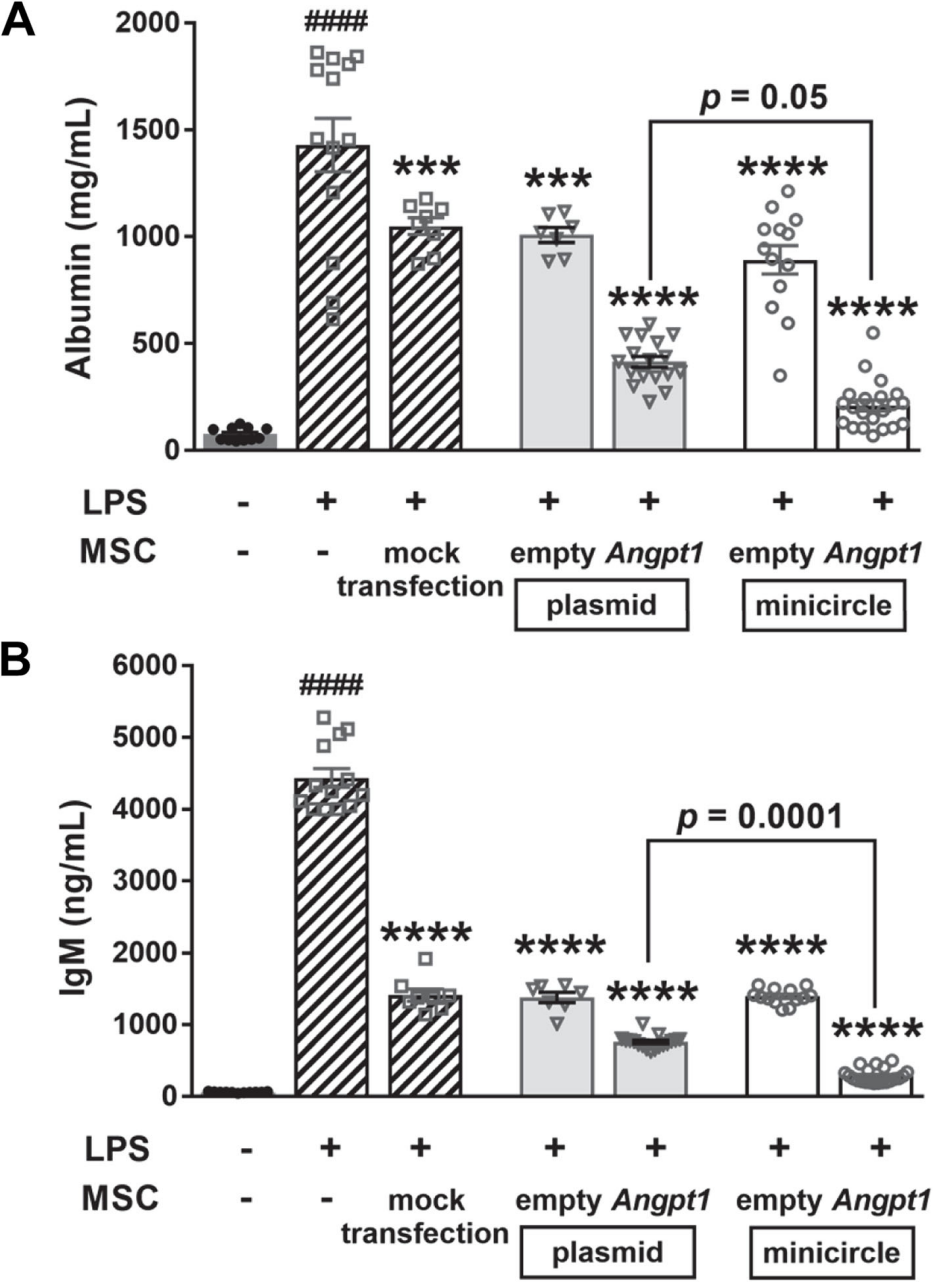
Effect of MSCs alone or ANGPT1-transfected (plasmid vs. MC) on LPS-induced pulmonary vascular permeability. Pulmonary vascular permeability was assessed by measurement of **a** albumin and **b** IgM in BAL fluids using ELISAs. Group comparisons were analyzed by one-way ANOVA with Bonferroni post hoc test. ^####^*p* < 0.0001, sham/vehicle vs. LPS/vehicle group. ***p < 0.001 and ****p < 0.0001, LPS/vehicle vs. each treated group

The LPS administration induced an increase in pulmonary vascular permeability due to the loss of integrity of the alveolar-capillary membrane barrier, which can be detected by measuring levels of high molecular weight proteins such as albumin (Fig. 4a) or IgM (Fig. 4b) in BAL fluid. Compared to sham mice (sham/vehicle group), mice challenged with LPS (LPS/vehicle group) showed significant increases in albumin and IgM (*p* < 0.0001). Mice treated with non- or mock-transfected MSCs showed a modest reduction in pulmonary vascular permeability, while mice receiving MSCs transfected with ANGPT1 (MSCs with pANGPT1 or MC-ANGPT1 transfection) showed a further reduction. However, MC-ANGPT1-transfected MSCs showed the greater reductions of albumin and IgG in BAL fluids compared to pANGPT1-transfected MSCs (*p* < 0.05 for albumin, *p* < 0.0001 for IgM; Fig. 4).

## Discussion

We have previously reported improved therapeutic efficacy of MSCs engineered to overexpress ANGPT1 using plasmid DNA in LPS-induced ALI in mice [9]. Here, we show that overexpression of ANGPT1 using minicircles can further reduce alveolar neutrophil infiltration, pulmonary inflammatory mediators, and lung vascular permeability compared to treatment using the traditional plasmid system.

To date, there is unfortunately no specific treatment or cure for ARDS patients. Clinically, treatment focuses on providing supportive care including (1) mechanical ventilation to ensure enough blood oxygenation, (2) prone positioning, (3) deep sedation with neuromuscular blockade to prevent unnecessary movement and/or agitation, (4) fluid management to prevent and/or reduce pulmonary edema, and (5) extracorporeal membrane oxygenation [30]. With extensive preclinical literature on the potential immunomodulatory actions of MSCs in animal models of acute injury, MSCs are now being considered as a potential therapy for ARDS. Indeed, several clinical trials have investigated the safety and efficacy of these cells in humans, including the START [14, 15] and MUST-ARDS trials [16].

In recent years, various strategies have been reported to increase the therapeutic potential of MSCs in vitro and in vivo. Pre-treatment of MSCs using hypoxia, pharmacological agents, and physical or chemical factors have been shown to improve MSC survival and even boost potency of MSCs in angiogenesis and immunomodulation [31]. However, the pre-treatment approach has the disadvantage of the effect being weakened or lost immediately after removal of the pre-treatment factor. In contrast, genetic modification of MSCs may offer a more persistent beneficial effect compared to the pre-treatment strategy. The overexpression or knockdown of specific genes has been used to enhance the therapeutic potential of MSCs by modulating survival, migration, adhesion, and regenerative and immunomodulatory effects [32]. Promising therapeutic benefits were also obtained after injection of MSCs in various preclinical models, such as heart and liver failure, blood production, neural injuries, sepsis, and cancer [33].

Plasmids are circular or linear extrachromosomal DNA elements found in genomes of prokaryotes [34]. Though it is an essential and ubiquitous tool applied in molecular biology, traditional plasmid itself has many limitations for use in genetic modification of MSCs for therapeutic purposes. The classical plasmid systems typically have low efficiency in transfecting primary cells with a shorter duration of transgene expression. Additionally, traditional plasmid has been reported to lead to unwanted inflammatory response due to its unmethylated bacterial backbone, which silences the transgenes and/or eliminates the plasmid from the cytoplasm [35].

To produce enhanced and sustained transgene expression, optimization of plasmid construction technology has been evolving. Modifications have been explored in promoter and enhancer elements, polyadenylation signals, and the removal of CpG motifs and antibiotic resistance genes [36]. The minicircle system described in the current manuscript is a plasmid-based, minimized DNA vector [25]. To obtain minicircles, recombination sequences are added between the transgene expression cassette and the bacterial backbone. Using a minicircle producer *E. coli* strain, the addition of L-Arabinose turns on the recombination process and eliminates the plasmid backbone. This process results in a significant removal of CpG motifs and a reduced size which, when used for transfection, can lead to less immunogenic responses and higher transfection efficiency [24, 25].

Indeed, minicircles have been described to have enhanced and prolonged transgene expression in quiescent cells when applied in vitro or in vivo, such as in airway cells [37]. Dad et al. used minicircle vector-mediated delivery of nucleases, such as Zinc finger nucleases (ZFNs) and transcription activator-like effector nucleases (TALENs), for genome edition in HEK 293T cells. They demonstrated that this approach was more efficient, safe, and less toxic than conventional plasmid [38]. In vivo delivery (muscle injection) of short hairpin RNA interference targeting prolyl hydroxylase-2 (shPHD2) using minicircle vectors, compared to vehicle and plasmid-treated mice, showed improved postischemic neovascularization after hindlimb ischemia [39]. Minicircles deployed with magnetofection were shown to achieve high, safe, and sustained transgene expression (up to 4 weeks) transfection levels in primary neural stem cells (NSC) in vitro compared to the plasmid system. Furthermore, NSC daughter cells, such as neurons and oligodendrocytes, also showed transgene expression in a minicircle-engineered model, which was not observed when using the conventional plasmid transfection system [40].

Altogether, improved MSC efficiency using a minicircle system is a promising approach for cell therapy. Nonetheless, in acute critical care, a cryopreserved allogeneic “off-the-shelf” MSC product would be a more logistically feasible approach, given that acutely ill patients (such as ARDS and septic patients) require rapid deployment of therapy to the bedside. We have previously described that a cryopreserved-thawed unmodified MSC product, compared to a cultured MSC product, exhibits a similar immunomodulatory potency in vitro and in polymicrobial septic animal [41]. More research is needed to optimize the cryopreservation of genetically modified MSC products to advance this type of next-generation MSC therapy.

## Conclusion

In the current study, we demonstrate for the first time that ANGPT1 encoded in MC-transfected MSCs, compared to a plasmid, can achieve a more efficient and sustained expression of ANGPT1 protein levels, leading to an enhanced therapeutic effect in rescuing ALI in an animal model. By overexpressing ANGPT1 in MSCs, we demonstrate that genetic modification of MSCs can significantly improve the therapeutic benefits of these cells, therefore leading to a promising therapeutic approach to treat ARDS. The robust effects of MC-MSCs on inflammatory mediators and permeability in the ALI model suggest this strategy represents an alternative to the conventional non-viral plasmid vectors on genetic modification of MSCs. Cell and gene therapy is an innovative and disruptive field and significant progress is underway. With advances to produce greater transgene expression, genetically modified MSCs could become the next generation of cell therapies.

## Data Availability

Not applicable.

## Abbreviations

ALI: Acute lung injury
ANGPT-1: Angiopoietin 1
ARDS: Acute respiratory distress syndrome
BAL: Bronchoalveolar lavage
IL-6: Interleukin-6
IFN-γ: Interferon gamma
LPS: Lipopolysaccharide
MC: Minicircle
MCP-1: Monocyte chemoattractant protein-1
MIP-2: Macrophage inflammatory protein-2
MSC: Mesenchymal stem cell
pANGPT1: Plasmid ANGPT1
shPHD2: Short hairpin RNA
TALENs: Transcription activator-like effector nucleases
TNF-α: Tumor necrosis factor alpha
VEGF: Vascular endothelial growth factor
ZFNs: Zinc finger nucleases
